# A Synthetic Modified Live Chimeric Marker Vaccine against BVDV-1 and BVDV-2

**DOI:** 10.3390/vaccines8040577

**Published:** 2020-10-02

**Authors:** Susanne Koethe, Patricia König, Kerstin Wernike, Florian Pfaff, Jana Schulz, Ilona Reimann, Birgit Makoschey, Martin Beer

**Affiliations:** 1 Institute of Diagnostic Virology, Friedrich-Loeffler-Institut, Federal Research Institute for Animal Health, 17493 Greifswald-Insel Riems, Germany; Susanne.Koethe@fli.de (S.K.); Patricia.Koenig@fli.de (P.K.); Kerstin.Wernike@fli.de (K.W.); Florian.Pfaff@fli.de (F.P.); ilona.reimann@t-online.de (I.R.); 2 Institute of Epidemiology Friedrich-Loeffler-Institut, Federal Research Institute for Animal Health, 17493 Greifswald-Insel Riems, Germany; Jana.Schulz@fli.de; 3 Intervet International B.V., MSD Animal Health, 5831 AN Boxmeer, The Netherlands; birgit.makoschey@merck.com

**Keywords:** BVDV, pestiviruses, DIVA vaccine, vaccination, challenge, CP7, chimera, N^pro^, E^rns^

## Abstract

Bovine viral diarrhea virus (BVDV), a pestivirus which exists in the two distinct species BVDV-1 (syn. *Pestivirus A*) and BVDV-2 (syn. *Pestivirus B*), is the causative agent of one of the most widespread and economically important virus infections in cattle. For economic as well as for animal health reasons, an increasing number of national BVDV control programs were recently implemented. The main focus lies on the detection and removal of persistently infected cattle. The application of efficient marker or DIVA (differentiation of infected from vaccinated animals) vaccines would be beneficial for the eradication success in regions with a high BVDV prevalence to prevent fetal infection and it would allow serological monitoring of the BVDV status also in vaccinated farms. Therefore, a marker vaccine based on the cytopathic (cp) BVDV-1b strain CP7 was constructed as a synthetic backbone (BVDV-1b_synCP7). For serological discrimination of vaccinated from infected animals, the viral protein E^rns^ was substituted by the heterologous E^rns^ of Bungowannah virus (BuPV, species *Pestivirus F*). In addition, the vaccines were attenuated by a deletion within the type I interferon inhibitor N^pro^ protein encoding sequence. The BVDV-2 vaccine candidate is based on the genetic sequence of the glycoproteins E1 and E2 of BVDV-2 strain CS8644 (CS), which were introduced into the backbone of BVDV-1b_synCP7_ΔN^pro^_E^rns^ Bungo in substitution of the homologous glycoproteins. Vaccine virus recovery resulted in infectious cytopathic virus chimera that grew to titers of up to 10^6^ TCID_50_/mL. Both synthetic chimera BVDV-1b_synCP7_ΔN^pro^_E^rns^ Bungo and BVDV-1b_synCP7_ΔN^pro^_E^rns^ Bungo_E1E2 BVDV-2 CS were avirulent in cattle, provided a high level of protection in immunization and challenge experiments against both BVDV species and allowed differentiation of infected from vaccinated cattle. Our study presents the first report on an efficient BVDV-1 and -2 modified live marker vaccine candidate and the accompanying commercially available serological marker ELISA system.

## 1. Introduction

Bovine viral diarrhea virus (BVDV) infection is one of the most important diseases of cattle stock worldwide with an enormous economic and animal welfare impact on beef and dairy industries [1]. In several European countries, a bovine viral diarrhea (BVD) eradication program was implemented [2,3,4] in the last decades. Surveillance of cattle herds by serological diagnostics and eradication of persistently infected (PI) animals with or without vaccination are the pillars of BVDV control [5]. However, marker vaccines would help to implement vaccination programs together with the ongoing eradication efforts, but those vaccines are not yet available.

BVDV has a single stranded positive-sensed RNA genome with a size of about 12.3 kilobases (kb) and belongs to the genus *Pestivirus* of the *Flaviviridae* family [6]. The genus *Pestivirus* is quite heterologous and an increasing number of new members has been identified in the past years [7,8,9,10,11,12]. Different mammals are susceptible to pestivirus infections, predominately ruminants and swine. BVDV is subdivided in two species, BVDV-1 (*Pestivirus A*) and BVDV-2 (*Pestivirus B*) [13], which are further divided in subtypes. Analysis revealed at least 21 subtypes within BVDV-1 and 4 within BVDV-2 [14]. Furthermore, both species occur in two biotypes, namely noncytopathic (ncp) and cytopathic (cp) viruses, according to their ability to induce apoptosis in infected cell culture [15]. Acute infections by both species can result in respiratory, enteric and reproductive disorders. Disease manifestations may range from subclinical or mild symptoms to fatal disorders, e.g., hemorrhagic syndrome (HS) and mucosal disease (MD) [16]. Infection of pregnant animals during the first trimester with ncp viruses of both species may cause fetal death or the birth of persistently infected (PI) calves [17]. These immunotolerant carriers shed livelong virus in high amounts and serve as the primary reservoir for BVDV in all cattle populations worldwide [14,18,19].

All pestiviruses share a similar genomic organization, facilitating the generation of chimeric viruses by the interchangeability of individual proteins. The RNA genome encodes four structural proteins (C, E^rns^, E1 and E2) and at least eight nonstructural proteins (N^pro^, p7, NS2, NS3, NS4A, NS4B, NS5A and NS5B) [6]. The immunodominant proteins of BVDV are the nonstructural protein NS3 and the glycoproteins E^rns^ and E2, which induce significant and detectable antibody titers in infected animals [20,21]. The generation of infectious virus chimera through substitution of genes, e.g., E2 and E^rns^ from a heterologous pestivirus without affecting the viability of the virus, has been described before [22,23,24]. The E2 protein mediates virus entry and acts as major immunogenic protein on the induction of neutralizing antibodies [25]. Moreover, E2 forms heterodimers with E1 [26], and determines species tropism [27]. The proteins N^pro^ and E^rns^ are unique to pestiviruses and mediate the evasion from the host’s IFN response and manipulation of the host’s self-non-self-discrimination for a successful establishment of persistent fetal infection and immunotolerance [28,29,30,31,32,33]. E^rns^ is an essential structural component of the viral particle [34], and its RNase activity is also antagonizing the host immune system. Furthermore, the abrogation of the RNase activity of *Pestivirus C* (syn. classical swine fever virus (CSFV)) through mutations lead to a virus attenuation in animal experiments [35]. N^pro^ was shown to be nonessential for most of the pestiviruses [36]. 

In 2003, the atypical pestivirus “Bungowannah virus” (BuPV) caused an outbreak of sudden deaths of piglets on a farm in New South Wales, Australia [7]. BuPV was associated with a high incidence of stillbirths in pigs and was identified as the causative agent of the porcine myocarditis syndrome [37]. However, no evidence of transmission into the neighboring environment or further regions was found [38]. The virus is circulating only in the initially affected farm complex. A high genetic stability of the RNA genome was monitored between 2010 and 2014 [39]. Pan-pesti reactive monoclonal antibodies were not able to detect BuPV antigen in infected cell cultures indicating the existence of major antigenic differences [40]. However, complementation studies demonstrated the compatibility of BuPV and BVDV envelope proteins [41]. 

In areas with a high density of cattle and a high BVDV seroprevalence or in free regions with a high risk of BVDV re-introduction, vaccination is an important pillar of BVDV control programs [42]. The major aim of BVDV vaccination is the effective prevention of transplacental infection of fetuses that may result in the birth of PI animals [43,44]. Furthermore, an efficient vaccine candidate should mediate cross protection against the common circulating subtypes of BVDV-1 and -2 [45]. 

Numerous inactivated as well as modified live vaccines (MLV) are currently commercially available. Mono- or bivalent vaccine formulations exist as single or combination vaccines against shipping fever and crowding disease, e.g., Pyramid^®^/Presponse^®^ family of vaccines (Boehringer Ingelheim Vetmedica GmbH, Ingelheim/Rhein, Germany), INFORCE^®^ (Zoetis Belgium SA, Louvain-La-Neuve, Belgium). An example of a bivalent BVDV-1/2 vaccine represents the MLV Bovela^®^ (Boehringer Ingelheim Vetmedica GmbH, Ingelheim/Rhein, Germany), which includes the ncp strains KE9 (BVDV-1b) and NY93 (BVDV-2a). Regarding the phenotypic and genetic stability of their attenuation, both strains are attenuated by double individual genomic deletions: N^pro^ codons 5 to 168 and E^rns^ codon 349 have been removed (citied from the CVMP assessment report for Bovela (EMEA/V/C/003703/0000)). In contrast to ncp BVDV, cp viruses are not able to establish a persistent infection in the fetus and are therefore preferably used in modern MLV. None of the current commercially available vaccines functions as marker vaccine and discrimination of vaccination from field virus infection by serological surveillance is so far not possible. They only serve as genetic DIVAs, meaning that the vaccine virus can be differentiated using RT-PCR and/or sequencing methods. 

A further disadvantage of the current live vaccines is the interference with the BVD ear notch diagnostics by their detection in tissue samples of newborn calves whose mothers were immunized within the first trimester of gestation [2,46]. Therefore, the need of safely attenuated and efficient BVDV marker vaccines is still in discussion.

Overall, the development of a bivalent vaccine against BVDV-1 and -2 that enables the DIVA principle by serological methods is of great importance for implemented eradication programs. The feasibility to develop a marker vaccine for pestiviruses, which is based on the substitution of viral protein, has been shown for CSFV. The Suvaxyn CSF Marker^®^ vaccine (Zoetis Belgium SA, Belgium), based on a live recombinant E2 gene deleted BVDV-1b (strain CP7) containing CSFV E2 protein, was market authorized in 2015. Efficacy as well as safety of this chimeric pestivirus vaccine against CSFV genotypes 1 and 2 has been confirmed in numerous vaccination challenge trials [47]. In line with glycoprotein E2, the likewise immunogenic E^rns^ protein is a promising marker candidate. The amino acid sequence identities within the E^rns^ proteins of different pestivirus species are less than 80% and the immunogenic regions are well characterized [48,49]. Chimeric BVDV containing glycoprotein E^rns^ of heterologous pestiviruses such as *Pestivirus G* (syn. giraffe pestivirus), reindeer pestivirus or *Pestivirus E* (pronghorn antelope pestivirus), have successfully been constructed and their potential as marker vaccines against BVDV infections has been evaluated [24]. 

Our study describes the construction of a completely synthetic BVDV-1 clone based on strain CP7 lacking the N^pro^ protein and expressing a heterologous E^rns^ protein of BuPV (E^rns^ Bungo) instead of the BVDV-E^rns^. Furthermore, the marker principle, which is defined by the absence of BVDV E^rns^-specific antibodies upon immunization and the protective capacity of the vaccination, was demonstrated in both BVDV-1 and BVDV-2 challenge trials with cattle. 

## 2. Materials and Methods 

### 2.1. Cells

All cell lines were obtained from the Collection of cell lines in veterinary medicine, CCLV, Friedrich-Loeffler-Institut (FLI), Greifswald-Insel Riems. The diploid bovine esophageal cell line KOP-R (RIE224) was used for electroporation of infectious RNA, virus rescue, virus propagation, virus isolation, growth kinetics and neutralization assays. Virus titrations were performed on Madin–Darby bovine kidney (MDBK) cells (RIE261). BuPV was grown on porcine kidney (SK6) (RIE262) cells. To perform the IFN bioassay, the recombinant SK6-MxLuc cell line was employed (RIE1272) (kindly provided by N. Ruggli, IVI, Bern, Switzerland) which expresses the firefly luciferase under control of the interferon inducible Mx promoter [50].

### 2.2. Viruses

CP7, a cp BVDV-1b strain, was isolated from a case of fatal Mucosal Disease [51]. The ncp BVDV-1b strain SE5508 was isolated from a healthy immunotolerant calf. SE5508 was used for challenge infections and neutralization assays as heterologous BVDV-1 strain. BVDV-2 strain CS8644, also a ncp phenotype, was isolated from a healthy immunotolerant calf in Germany [52]. The challenge strain, ncp BVDV-2a HI916, was isolated in 2005 during an acute outbreak of BVD in Germany [53]. The atypical porcine pestivirus BuPV was isolated from piglets that suffered from sudden deaths on a farm in New South Wales, Australia in 2003 [7].

### 2.3. Generation of Completely Synthetic CP7 Viruses and Virus Recovery

The infectious cDNA clones pBVDV-1b_synCP7 (synCP7) and pBVDV-1b_synCP7_ΔN^pro^ (synCP7_ΔN^pro^) were constructed based on the published sequence of the cytopathic BVDV isolate CP7 (BVDV-1b strain) (GenBank accession number: BVU63479). Five synthetic DNA fragments (Geneart AG, Regensburg, Germany) were ligated into plasmid vector pA [54] to obtain completely synthetic clones. In construct synCP7_ΔN^pro^, most of the N^pro^ encoding genomic region was deleted (nucleotides (nt) 422–889, amino acids (aa) 13–168) [53]. Construct pBVDV-1b_synCP7_ΔN^pro^_E^rns^ Bungo (synCP7_ΔN^pro^_E^rns^ Bungo) was generated by substitution of the E^rns^ protein encoding genomic region of synCP7_ΔN^pro^ with BuPV-E^rns^ (GenBank accession number: NC_023176). In comparison to CP7-E^rns^ (227 aa), BuPV-E^rns^ (E^rns^ Bungo) (222aa) is lacking seven aa in the transporter peptide region at the C-terminus. To ensure correct processing of the BuPV-E^rns^ protein and the downstream localized E1 protein within the polyprotein of synCP7_ΔN^pro^_E^rns^ Bungo, the C-terminus of CP7-E^rns^, which harbors the transporter peptide region [55], was retained (aa 192–227). To obtain the infectious DNA clone pBVDV-1b_synCP7_ΔN^pro^_E^rns^ Bungo_E1E2 BVDV-2 (synCP7_ΔN^pro^_E^rns^ Bungo_E1E2CS), the E1 and E2 encoding genomic region of CP7 was substituted within plasmid pBVDV-1b_synCP7_ΔN^pro^_E^rns^ Bungo with E1 and E2 of BVDV isolate CS8644, a BVDV type-2 strain. All substitutions were done by Fusion PCR methods [56] (details on request).

Plasmids were amplified in *Escherichia coli* DH10B^TM^ cells (Thermo Fisher Scientific, Darmstadt, Germany). Plasmid DNA was purified using Qiagen Plasmid Midi Kit (Qiagen, Hilden, Germany). The sequence was confirmed using Sanger sequencing. To recover virus, the cDNA construct was linearized by *SmaI* digest and in vitro transcribed by T7 RiboMax Large Scale RNA Production System (Promega, Walldorf, Germany). The resulting RNA was electroporated into bovine esophagus cells (KOP-R) [57]. The supernatant of electroporated cells was titrated to test for the presence of infectious virus. Recovered virus was serially passaged on KOP-R cells.

### 2.4. Growth Kinetics

To perform in vitro growth kinetics analyses, KOP-R cells were inoculated with various viruses at a multiplicity of infection (m.o.i.) of 1. Supernatants were collected at 0, 24, 48 and 72 h post-infection (p.i.) and titrated on MDBK cells. The titers were calculated and expressed as 50% tissue culture infective dose per ml (TCID_50_/mL). Growth kinetics were performed three times. Mean values from the biological replicates were calculated.

### 2.5. IFN Type I Reporter Assay Using Luciferase as Genetic Reporter

The IFN-a/b activity was measured by using firefly luciferase expressing porcine kidney cells (SK6-MxLuc). Briefly, a total of 1.5 × 10^5^ KOP-R cells was inoculated with the indicated viruses at an m.o.i. of 1. Two hours p.i., cell culture supernatants were discarded, cells were washed twice with PBS and 1.0 mL of culture medium was added. Supernatants were collected at 0, 24 and 48 h p.i. The UV-inactivated supernatants were applied to SK6-MxLuc cells and incubated for 24 h at 37 °C. Supernatants of mock-infected KOP-R cells were used as negative controls. Type I interferon in the supernatants acts on stimulation of the Mx-promoter and subsequent expression of luciferase activity. The measurement of firefly luciferase activity (bovine IFN-a/b) was carried out by using the Bright-Glo^TM^ Luciferase Assay System (Promega). Thereby, the Mono-oxygenation of luciferin is catalyzed by firefly luciferase and bioluminescence can be measured with a luminometer (TECAN infinite F200 PRO, Tecan Germany GmbH, Crailsheim, Germany). The IFN type I induction was calculated in relation to values of the negative control. Two biological and three technical replicates were performed.

### 2.6. Ethics, Animals and Experimental Design

The animal study was conducted in compliance with governmental animal welfare guidelines and regulations and was approved by the State Office of Agriculture, Food safety, and Fishery in Mecklenburg-Western Pomerania under the registration number 7221.3-1-034/15.

Thirty conventionally reared female Holstein–Friesian calves obtained from local farms were enclosed in the vaccination and challenge infection study after being tested negative for the presence of BVDV- and bovine herpes virus (BoHV)-1-specific antibodies. The animals were allocated into six different groups for the trial with a group size of five animals each. The animals were housed in the biosafety level-2 facility of the FLI. Their age ranged from four to seven months with an even distribution in each group. Animals were vaccinated once or twice at an interval of four weeks. Four weeks after the final immunization, both groups as well as the non-vaccinated control group were intranasally infected with the respective BVDV -1 or -2 challenge strains. For BVDV-1 vaccination, the virus stocks were adjusted to 1 × 10^6^ TCID_50_ synCP7_ΔN^pro^_E^rns^ Bungo. The calves of the BVDV-1 trial were vaccinated intramuscularly (i.m.) with 1 mL containing 5 × 10⁵ TCID_50_. Back titrations verified in the first vaccination round (V1) at day 0 a dose of 5.15 × 10^5^ TCID_50_ and 3 × 10^5^ TCID_50_ in the second round (V2) at day 28. For the intranasal BVDV-1 challenge infection at day 56, 2 mL (1 mL per nostril) with 2 × 10^7^ TCID_50_ of the SE5508 strain were applied.

The calves of the BVDV-2 trial were vaccinated i.m. with 1 mL containing 1 × 10^6^ TCID_50_ synCP7_ΔN^pro^_E^rns^ Bungo_E1E2CS. After back titration, an actual first dose of 3.41 × 10^6^ TCID50 (V1) at day 0 were confirmed and a respective second dose of 1.47 × 10^6^ TCID_50_ (V2) at day 28. For the intranasal BVDV-2 challenge infection at day 56, 2 mL (1 mL per nostril) of the HI916 strain with 2 × 10^5^ TCID_50_ was inoculated. The back titration revealed a titer of 3.3 × 10^5^ TCID_50_. Due to the enhanced virulence of isolate HI916, the challenge virus dose was reduced compared to the BVDV-1 trial. All viral suspensions were back titrated on MDBK cells to confirm the infectious titer of the respective inocula.

### 2.7. Clinical Evaluation

During the course of the trials, body temperatures were measured daily and clinical examinations were carried out daily. The calves were also examined for adverse reactions immediately after vaccination and challenge infection. Signs of clinical disease, focusing on respiratory and digestive disorders, and the general health status (depression, feed intake and behavior) were controlled. Cumulative clinical scores were defined by ranking respiratory, enteric and common signs from 0 (inconspicuous) to 4 (markedly abnormal).

### 2.8. Nasal Swabs and EDTA-Blood Samples

Nasal swabs and EDTA-blood samples were collected daily over a period of 10 days after the respective first vaccination and for 14 days after the challenge infection. Serum samples were taken weekly for monitoring the serological responses. The samples were subjected to hematological, virological and serological investigations. 

### 2.9. Hematological Investigations 

Blood samples were taken by jugular venipuncture and collected in potassium EDTA coated sterile blood collection tubes (Monovette) (Sarstedt, Nümbrecht, Germany). Differential white blood cell counts were determined using Abbott CellDyn 3700 analyzer (Abbott GmbH, Wiesbaden, Germany).

### 2.10. Virus Isolation

Virus isolation in cell culture was essentially performed as described by Zemke et al. [53]. Shortly, virus isolation from nasal swabs and EDTA-blood was conducted. A monolayer of KOP-R cells was inoculated with four replicates per animal and specimen. The virus replication was controlled by indirect immunofluorescence staining of the viral NS3 protein (mab mix WB103/105 from c.c.pro GmbH, Oberdorla, Germany). Binding was detected using the Alexa488 goat anti-mouse IgG conjugate (Thermo Fisher Scientific). Evaluation was carried out using the fluorescence microscope Nikon Eclipse Ti (Nikon, Düsseldorf, Germany). One blind passage of supernatants was performed after 3–4 days of inoculation.

### 2.11. Serology 

For weekly intravenous serum sampling, sterile blood collection tubes (Monovette) with clot activator were used. After centrifugation at 3000 rpm, serum aliquots were stored at −20 °C. For serological investigation, all sera were inactivated at 56 °C for 30 min.

The serological investigation was conducted using the ID Screen^®^BVD p80 antibody competition ELISA (IDVet, Grabels, France) and Monoscreen Ab ELISA BVDV (E0) (Bio-X Diagnostics S.A., Rochefort, Belgium). The samples were processed following the manufacturer´s instructions. For the ID Screen^®^BVD p80 antibody competition ELISA, the relative blocking values (%S/N) were calculated and samples with %S/N ≤ 40% were considered as positive, between 40% and 50% as suspicious and ≥50% as negative as recommended by the manufacturer. For this study, all suspicious (intermediate) test results were classified as positive. For the Monoscreen Ab ELISA BVDV (E0) ELISA, all % inhibition E^rns^ values were calculated and samples with % inhibition E^rns^ values ≥50% were considered as positive as recommended by the manufacturer.

Sera from all animals were also tested in a standard neutralization assay (NA) against homo- and heterologous BVDV-1 (CP7 and SE5508) and BVDV-2 (CS8644 and HI916). The NA was conducted as described by Zemke et al. [53]. Titers were expressed as reciprocal of the highest dilution that caused 50% neutralization per ml (ND_50_/mL).

### 2.12. Sequence Analysis 

All constructed vaccine viruses and passaged viruses were checked for correct genomic arrangement and nucleotide sequences based on the complete genome using Sanger sequencing. Viral RNA was extracted from infected cell culture supernatants using the QIAmp Viral RNA Mini Kit (Qiagen, Germany) according to the manufacturer´s instructions. The complete genome was amplified using several primers evenly distributed and overlapping along the genome using Superscript III One-Step RT-PCR system (Thermo Fisher Scientific, Germany). The RT-PCR products were purified by agarose gel electrophoresis and subsequent gel extraction using the QIAquick Gel Extraction Kit (Qiagen, Germany). Sequencing of the amplified part of the genome was carried out using BigDye^®^Terminator v1.1 Cycle sequencing kit (Applied Biosystems, Thermo Fisher Scientific) and a Genetic Analyzer 3130XL (Applied Biosystems). Nucleotide sequences were analyzed using the Geneious prime software (version 2019.2.3; Biomatters, Auckland, New Zealand). Custom primers were used for amplification and sequencing (Metabion, Germany and Eurofins, Germany; details on request).

Passaged virus and the re-isolated BVDV-1b_synCP7_ΔN^pro^_E^rns^ Bungo_E1E2CS vaccine virus from a single animal (#474) from purified leukocytes (d5) after the first vaccination were checked for genomic arrangement and nucleotide sequences based on the complete genome using Illumina high-throughput sequencing. Briefly, total RNA was extracted from the supernatant of infected cell culture using Trizol lysis reagent (Thermo Fisher Scientific) in combination with the RNeasy Mini kit (Qiagen) and on-column DNase digestion using an RNase-free DNase kit (Qiagen). Subsequently, double-stranded cDNA was generated using the cDNA Synthesis System (Roche, Mannheim, Germany) in combination with random hexanucleotides (Roche) and was then fragmented to an average size of about 300 base-pairs (bp) using a M220 ultrasonicator (Covaris, Woburn, MA, USA). The fragmented cDNA was then transformed into barcoded sequencing libraries using the SPRI-TE instrument with SPRIworks II cartridges (Beckman Coulter GmbH, Krefeld, Germany) along with appropriate adapters. Resulting libraries were quantified using the Kapa Library Quantification kit (Kapa Biosystems Inc., Wilmington, MA, USA) and subsequently sequenced on the Illumina MiSeq platform using a MiSeq reagent kit, version 3 (Illumina, San Diego, CA, USA) in 2 × 300 bp paired end mode according to the manufacturer’s instructions.

Raw read data was subsequently mapped to appropriate BVDV-1, BVDV-2 and BuPV reference sequences and matching reads were assembled de novo using the 454 Sequencing Systems Software suite (version 3.0; Roche). The resulting full genome sequences were manually checked using Geneious Prime software (version 2019.2.3; Biomatters Ltd., Auckland, New Zealand).

### 2.13. Statistics

Statistical analysis was performed to investigate differences in the growth kinetics among the control, the synthetic CP7 viruses, and BuPV. A linear mixed model was used to investigate the group effects on the log transformed titers accounting for the random effect of the three replicates in each group and the repeated measures at 0, 24, 48 and 72 h p.i.

The cumulated daily scoring values resulting from virological investigations of nasal swabs and EDTA-blood samples in the challenge infection study were compared by pairwise Wilcoxon tests with Bonferroni correction for multiple testing (uncorrected level of significance 0.05). 

Statistical analysis was performed in R (version 4.0.0) (The R Foundation for Statistical Computing, open source project).

## 3. Results

### 3.1. Construction and Characterization of BVDV-1b_synCP7_ΔN^pro^_E^rns^ Bungo and BVDV-1b_synCP7_ΔN^pro^_E^rns^ Bungo_E1E2 BVDV-2 

**Figure 1:**
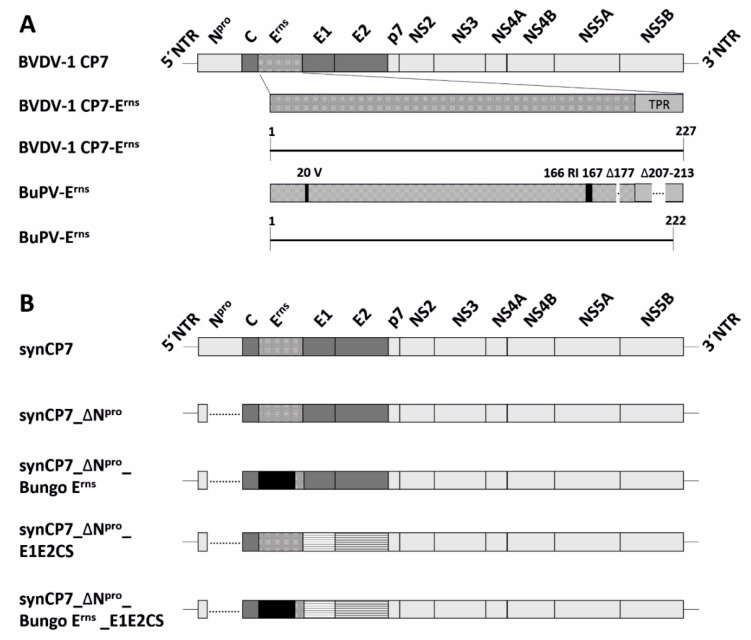
Schematic representation of bovine viral diarrhea virus (BVDV)-1-E^rns^ and Bungowannah virus (BuPV)-E^rns^ and the overview of synthetic recombinant viruses based on the synCP7. (**A**) Comparison of BVDV-E^rns^ (aa 227) and BuPV-E^rns^ (aa 222) protein. The numbering of amino acids in BuPV-E^rns^ correspond to BVDV-E^rns^. Insertions of amino acids in BuPV-E^rns^ are depicted as black bars and deletions as white bars in comparison to BVDV-E^rns^. NTR—non-translated region, TPR-transporter peptide region. (**B**) Schematic overview of the synthetic recombinant viruses based on the synCP7 backbone are shown.

Full synthetic BVD viruses based on the genetic background of the cytopathic BVDV-1b strain CP7 (synCP7) were constructed to obtain efficient innocuous marker vaccine candidates. To maintain comprehensive vaccine safety, the viral IFN antagonist N^pro^ was deleted with retaining only the first 12 aa to ensure a correct processing of the polyprotein (synCP7_ΔN^pro^). Furthermore, to discriminate vaccinated from field infected animals the genetic sequence of the immunogenic E^rns^ protein of BVDV was replaced by the E^rns^ encoding genome region of BuPV (synCP7_ΔN^pro^_E^rns^ Bungo). Overall, the E^rns^ protein from BuPV bears three additional aa (aa 20, 166, 167 (BVDV-1 numbering)) that are not present in E^rns^ BVDV and one aa deletion (aa 177). Furthermore, the transporter peptide region (TPR) at the C-terminus of BuPV_E^rns^ is seven aa shorter than the BVDV E^rns^ (aa207–213) (Figure 1A). The TPR of BVDV was retained (192–227aa) in synCP7_ΔN^pro^_E^rns^ Bungo to ensure correct E^rns^ and downstream E1 protein processing. Secondly, the E1E2 proteins of CP7 were substituted with E1E2 of the BVDV-2 strain CS4688 (synCP7_ΔN^pro^_E^rns^ Bungo_E1E2CS). In order to enable analysis of a possible impact of the E^rns^ substitution in the context of synCP7_ΔN^pro^_E^rns^ Bungo_E1E2CS in vitro, also synCP7_ΔN^pro^_E1E2CS, expressing the homologous CP7-E^rns^ (BVDV-1), was generated (Figure 1B).

After initial virus rescue by RNA transfection, the viruses synCP7, synCP7_ΔN^pro^, synCP7_ΔN^pro^_E^rns^ Bungo, synCP7_ΔN^pro^_E1E2CS and synCP7_ΔN^pro^_E^rns^ Bungo_E1E2CS were passaged on KOP-R cells. The expression of BuPV-E^rns^ and E2 of BVDV-2 were confirmed in infected cells by immunofluorescence staining using appropriate specific antibodies.

**Figure 2:**
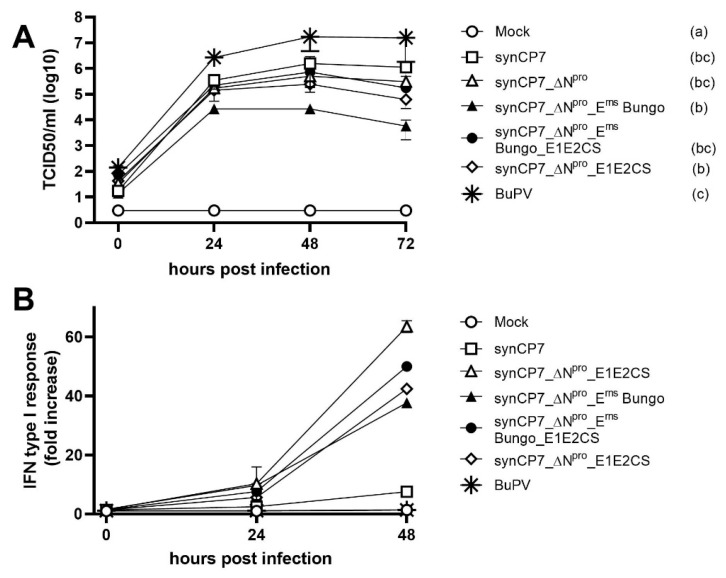
In-vitro characterization of BVDV-1b synCP7_ΔN^pro^_E^rns^ Bungo and synCP7_ΔN^pro^_E^rns^ Bungo_E1E2CS. (**A**) Single step growth kinetics in KOP-R cells at an m.o.i. of 1. Supernatants were harvested 0, 24, 48 and 72 h p.i. and titrated on Madin–Darby bovine kidney (MDBK) cells. Mean values of three representative experiments are displayed. Error bars represent standard deviations. Letters in brackets (a, b, bc, c) display the results of all pairwise comparisons. Viruses sharing the same letter did not show significantly different titers over time. (**B**) Determination of IFN type I response after virus infection. The induction of IFN was determined using an IFN bioassay based on an Mx promotor reporter system with luciferase. KOP-R cells were inoculated with the respective virus at an m.o.i. of 1. Supernatants were harvested 0, 24 and 48 h p.i., UV-inactivated supernatants were applied to SK6-MxLuc reporter cells and incubated for 24 h at 37 °C. The IFN type I induction was related to the values of the control. Mean values of two representative experiments are displayed. Error bars represent standard deviations.

Growth kinetics of the recovered viruses were performed using an m.o.i. of 1 (Figure 2A) in KOP-R cells. Virus titers were determined at 0, 24, 48 and 72 h p.i.. In comparison to wild type-like BVDV-1b_synCP7 and ΔN^pro^-mutants, synCP7_ΔN^pro^_E^rns^ Bungo was less efficient in cell culture growth resulting in lower virus titers independent of the time point. Whereas, synCP7_ΔN^pro^_E^rns^ Bungo_E1E2CS reached titers that were comparable to synCP7_ΔN^pro^-mutants and independent from the origin of the viral E^rns^. BuPV was included in the analysis as a control.

The unique pestivirus proteins N^pro^ and E^rns^ are involved in the modulation of the IFN type I response of the host. The loss of IFN type I repression can be regarded as a step towards attenuation and vaccine safety. To test the production of IFN type I in response to an in vitro infection of KOP-R cultures, IFN type I reporter cells, SK6 MxLuc, were stimulated with inactivated supernatants of KOP-R cultures infected with synCP7, synCP7_ΔN^pro^, synCP7_ΔN^pro^_E^rns^ Bungo, synCP7_ΔN^pro^_E1E2CS and synCP7_ΔN^pro^_E^rns^ Bungo_E1E2CS collected 0, 24 and 48 h p.i.. The inducibility of IFN type I was quantified by luciferase expression measured as relative light units (RLU). In general, the supernatant of infected cells collected at 48 h p.i. revealed the highest capacity to induce IFN type I in comparison to 0 or 24 h p.i.. SynCP7 and BuPV were able to suppress the induction of IFN type I response, whereas the deletion of IFN antagonist N^pro^ resulted in a markedly increased IFN response (Figure 2). Compared to the control (mock-infected cells), synCP7 and BuPV, IFN type I response was up to approximately 50-fold higher for all N^pro^ deletion mutants. The substitution of BVDV-E^rns^ with BuPV-E^rns^ in synCP7_ΔN^pro^_E^rns^ Bungo had only a minor impact on the induction of IFN type I (Figure 2B). As expected, the substitution of E1E2 of BVDV-1 CP7 by E1E2 of BVDV-2 CS8644 had no influence on the IFN type I response (Figure 2).

To determine the genetic stability of the rescued recombinant viruses, total RNA was prepared after at least 10 serial passages in interferon-competent cells and the viral full-length genome sequences were determined by Sanger sequencing and high-throughput sequencing approaches. RNA of BVDV-1b_synCP7_ΔN^pro^_E^rns^ Bungo was isolated from passage number 13 and 20. A single silent point mutation was detected in virus passage 13 within the E1 gene. This mutation could be confirmed also after 20 passages and furthermore, sequence analysis revealed two additional silent mutations in the genes of BuPV-E^rns^ and NS5B. The sequence of synCP7_ΔN^pro^_E^rns^ Bungo_E1E2CS8644 was stable over 10 passages in cell culture and no mutations could be detected.

### 3.2. BVDV-1b synCP7_ΔN^pro^_E^rns^ Bungo and BVDV-1b synCP7_ΔN^pro^_E^rns^ Bungo_E1E2 BVDV-2CS Vaccination of Cattle

**Figure 3:**
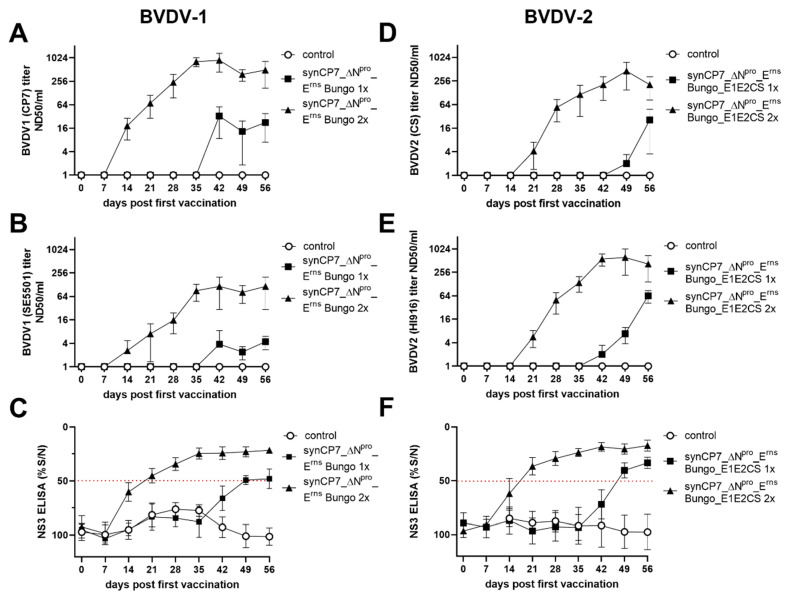
Antibody titer development after vaccination with BVDV-1b_synCP7_ΔN^pro^_E^rns^ Bungo and synCP7_ΔN^pro^_E^rns^ Bungo_E1E2CS. Neutralizing antibody titers after one (1x) (28 days post first vaccination) or two (2x) (0 and 28 days post first vaccination) shots of BVDV-1b_synCP7_ΔN^pro^_E^rns^ Bungo vaccination against the homologous strain BVDV-1 CP7 (**A**), the heterologous (challenge) strain BVDV-1 SE5501 (**B**), and NS3-specific antibodies in an ELISA (**C**) were determined. Titers of neutralizing antibodies after one (1x) (28 days post first vaccination) or two (2x) (0 and 28 days post first vaccination) shots of BVDV-1b_synCP7_ΔN^pro^_E^rns^ Bungo_E1E2CS vaccination against the homologous strain BVDV-2 CS8644 (**D**), the heterologous (challenge) strain HI916 (**E**), and titers of NS3-specific antibodies in a competitive NS3 (p 80) ELISA (**F**) were characterized. Mean group values and standard deviations are shown. (**C**,**F**) The dotted line marks the cut-off value of the NS3 ELISA.

To test the potential of BVDV-1b_synCP7_ΔN^pro^_E^rns^ Bungo and synCP7_ΔN^pro^_E^rns^ Bungo_E1E2CS as new BVDV marker vaccine candidates, one (1x) or two (2x) shots of the candidate vaccines were administered i.m.. Upon the first and second (at day 28) vaccination with BVDV-1b_synCP7_ΔN^pro^_E^rns^ Bungo, no virus could be re-isolated from nasal swabs or purified leukocytes. The rectal temperature and the leukocyte counts remained stable and no adverse reactions or clinical symptoms like diarrhea or nasal discharge were observed. To characterize the quantity of BVDV-specific antibodies produced upon vaccination, titers of serum E2-neutralizing (SN) antibodies against the homologous CP7 and a heterologous (challenge) SE5508 BVDV-1 strain were specified and NS3-specific antibodies were determined in a commercially available ELISA assay. The vaccination with BVDV-1b_synCP7_ΔN^pro^_E^rns^ Bungo resulted in the detection of BVDV-1b-specific E2-neutralizing antibodies with titers of up to 1:240 against the homologous CP7 strain and also NS3-specific antibodies. The second vaccination resulted in a marked booster of the antibody levels with titers of up to 1:889 (Figure 3A–C).

Upon the first and second vaccination with BVDV-1b_synCP7_ΔN^pro^_E^rns^ Bungo_E1E2CS, the rectal temperature remained stable and only non-specific clinical symptoms like nasal discharge or conjunctivitis on the pre-vaccination level were observed. In contrast to the BVDV-1 marker vaccination, virus could be re-isolated from one animal (#474) from purified leukocyte (d5) after the first vaccination. Sequencing analysis of the re-isolated and three times passaged BVDV-1b_synCP7_ΔN^pro^_E^rns^ Bungo_E1E2 BVDV-2 revealed two silent mutations, one within the CP7-E^rns^ transporter peptide, one in the BVDV-2 CS-E2 encoding genomic region and one amino acid substitution within the NS2 encoding genomic region. As shown for BVDV-1b_synCP7_ΔN^pro^_E^rns^ Bungo, also upon vaccination with BVDV-1b_synCP7_ΔN^pro^_E^rns^ Bungo_E1E2CS, serum neutralizing E2-BVDV-2-specific antibodies against the homologous CS8644 and a heterologous (challenge) HI916 virus strain in line with NS3-specific antibodies were detectable upon 14 to 21 days post vaccination with titers of up to 1:54 (Figure 3D–F).

### 3.3. BVDV-1 Challenge Infection of BVDV-1b_synCP7_ΔN^pro^_E^rns^ Bungo Vaccinated Cattle

**Figure 4:**
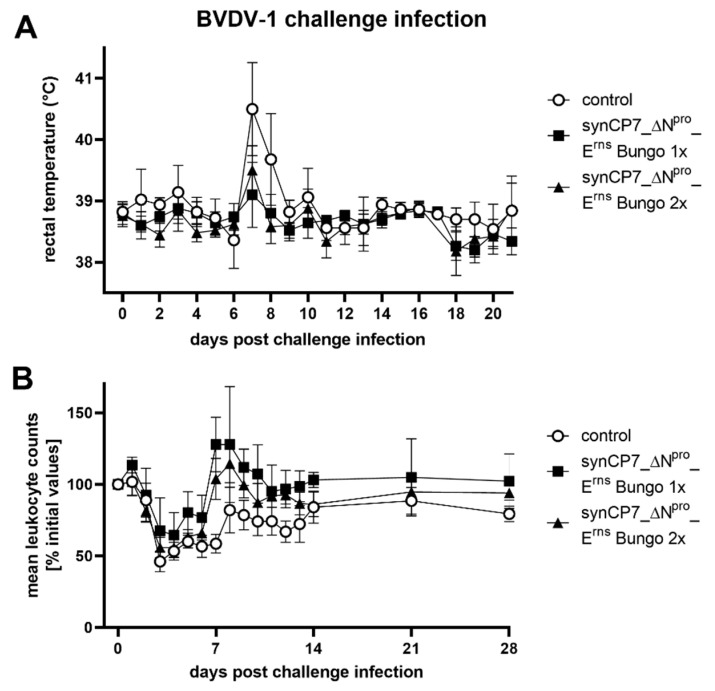
Characterization of the rectal body temperature and the course of blood leukocytes after BVDV-1 challenge infection of BVDV-1b_synCP7_ΔN^pro^_E^rns^ Bungo-vaccinated cattle. The animals were vaccinated with one (1x) or two (2x) shots of the candidate vaccine. (**A**) Mean rectal body temperature after the challenge infection. Temperatures lower than 39.5 °C are considered as physiological temperature, higher than 40 °C as fever. (**B**) Course of blood leukocyte counts after the challenge infection using EDTA blood as sample matrix. Mean values of the different groups are shown in percentage of the initial values (number of leukocytes measured at the day of challenge infection). The initial values (measured value at day of challenge) were set to 100%. Error bars represent standard deviations.

At day 56 of the trial, three groups of five animals each, the unvaccinated control and once and twice vaccinated animals, were infected with the heterologous BVDV-1 strain SE5508 intranasally (i.n.). All unvaccinated control animals developed typical and clear clinical signs of infection. The body temperatures showed a monophasic rise at day 7 post challenge infection (p.chall.) with a maximum mean group value of 40.5 °C (Figure 4A). In contrast, BVDV-1b_synCP7_ΔN^pro^_E^rns^ Bungo-vaccinated animals showed on day 7 p.chall. a moderate elevation of the body temperatures within the physiological temperature range, peaking at 39.1 °C (1x vaccinated) and 39.5 °C (2x vaccinated). In addition to the elevated body temperature, only minor additional clinical effects were observed after challenge infection. The control animals showed a slightly reduced feed intake and a marginally reduced general condition for one day (7 or 8 d p.chall.). Diarrhea was not observed and respiratory distress did not exceed the pre-challenge scores.

Furthermore, a severe leukopenia was determined in all control animals. The triphasic decrease (3, 6 and 12 d p.chall) in leukocyte counts showed a maximum mean level in reduction of 54% on day 3 p.chall. (Figure 4B). In line with the clinical observation, the triphasic decrease in leukocyte counts was less severe in the vaccinated compared to the control group. Nevertheless, differences between the vaccinated groups were detectable, as the drop of leukocytes was more, but not significantly, pronounced in the 2x than in the 1x vaccinated group. Moreover, a rebound in leukocyte counts was detected for the vaccinated animals as early as by day 7 and 8 p.chall., but not for the control group. The maximum mean decreases for the one shot vaccinated group was 35% (4 d p.chall.) and 44% for the two shots vaccinated animals (3 d p.chall.), respectively.

During the acute infection with BVDV-1, a transient viremia and nasal shedding may occur [16,58]. Therefore, virus isolation from nasal swabs and from blood was conducted daily from day 1 p.chall. until 14 days p.chall. A long and pronounced nasal shedding of challenge virus and viremia was found in all control animals. Virus was secreted from the nose on five to eight consecutive days, whereas virus was isolated from nasal swabs on 0–3 coherent days in the 1x vaccinated and on only 0–2 days in the 2x vaccinated group. Furthermore, virus was isolated over a period of 11 days from the leukocytes in the control group but only on one or two consecutive days in a few animals of the vaccinated groups, respectively (Table 1). Overall, the nasal shedding and the viremia were significantly reduced in both vaccinated groups compared to the unvaccinated control animals (*p* < 0.017).

### 3.4. BVDV-2 Challenge Infection of BVDV-1b_synCP7_ΔN^pro^_E^rns^ Bungo_E1E2 BVDV-2CS-Vaccinated Cattle

**Figure 5:**
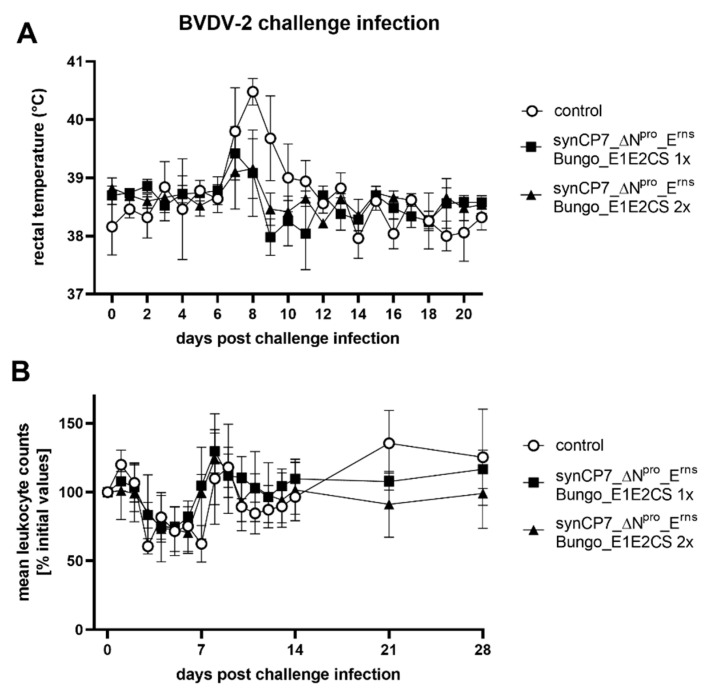
Characterization of the rectal body temperature and the course of blood leukocyte counts after BVDV-2 challenge infection of BVDV-1b_synCP7_ΔN^pro^_E^rns^ Bungo_E1E2CS-vaccinated cattle. The cattle were vaccinated with one (1x) or two (2x) shots of the candidate vaccine. (**A**) Course of mean rectal body temperature after the challenge infection - values lower than 39.5 °C are considered as physiological temperature, higher than 40 °C as fever. (**B**) Course of blood leukocyte counts after the challenge infection using EDTA blood. Mean values of the several groups are shown in percentage of the initial values. The initial values were set to 100% prior to challenge infection. Error bars represent standard deviations.

**Figure 6:**
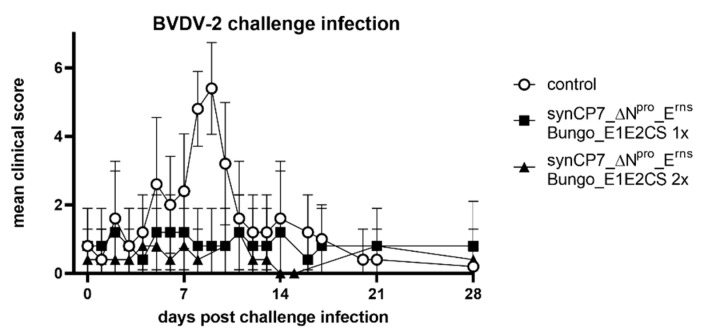
The clinical score after BVDV-2 challenge infection in BVDV-1b_synCP7_ΔN^pro^_E^rns^ Bungo_E1E2CS-vaccinated cattle. The cattle were vaccinated with one (1x) or two (2x) shots of the candidate vaccine. Each animal was examined daily and a clinical score considering respiratory and enteric disorder as well as common health and feed intake was determined. Mean group values are depicted. Error bars represent standard deviations.

All animals of the BVDV-1b_synCP7_ΔN^pro^_E^rns^ Bungo_E1E2CS vaccination groups and five unvaccinated control animals were challenged with the heterologous BVDV-2 strain HI916 i.n. on day 56 of the trial and developed in contrast to the BVDV-1 trial typical and clear clinical signs of infection in the following days. The body temperatures of the unvaccinated control animals showed a monophasic rise peaking on day 8 p.chall. with a maximum mean group value of 40.5 °C (Figure 5A). In contrast, vaccinated animals showed a reduced elevation in the body temperature on day 7 or 8 p.chall. staying in the physiological temperature range, peaking at 39.4 °C (1x vaccinated) and 39.2 °C (2x vaccinated). The clinical symptoms like conjunctivitis, pronounced nasal discharge, diarrhea, coughing and the loss of appetite were observed in the unvaccinated control group on day 7 to 10 p.chall., but were absent in the vaccinated groups. This is in line with the detection of fever in the control animals (Figure 6). Furthermore, a severe leukopenia was observed in all unvaccinated control animals. The triphasic decrease (3, 7 and 11 d p.chall.) in leukocyte counts showed a maximum mean level in reduction of 54% on day 3 p.chall. (Figure 5B). This is in contrast to the vaccinated groups, where the triphasic decrease in leukocyte counts was less severe and the following rebound in leukocyte numbers was observed markedly earlier on day 7 and 8 p.chall. compared to the unvaccinated control group. Only marginal differences between the vaccinated groups were detectable, as the drop of leukocytes was more pronounced in the 2x than in the 1x vaccinated group. The maximum mean decrease for the one shot vaccinated group was 26% (5 d p.chall.) and 30% for the two shots vaccinated animals (4–6 d p.chall.), respectively.

Significant clinical signs were only observed in the control group after virulent BVDV-2 challenge infection (Figure 6).

In contrast to the long and distinct challenge virus nasal shedding and viremia of the control animals, a very clear reduction in duration and virus titers was observed in the vaccinated cattle. Virus could be isolated from nasal swabs for up to nine consecutive days in the unvaccinated control group, whereas virus was isolated from nasal swabs of a single animal on three days in the 1x vaccinated and on one day each in two animals of the 2x vaccinated group. Moreover, the virus isolation from leukocytes was successful in the control group on 9–13 days in comparison to one to four days in both vaccinated groups (Table 2). Overall, the nasal shedding and the viremia were significantly reduced in both vaccinated groups compared to the unvaccinated control animals (*p* < 0.017).

### 3.5. Marker Serology

The marker principle of the candidate vaccines is based on the absence of detectable BVDV-E^rns^-specific antibodies in a commercial BVDV-specific ELISA assay upon vaccination and the consecutive BVDV-E^rns^-seroconversion following challenge infection.

**Figure 7:**
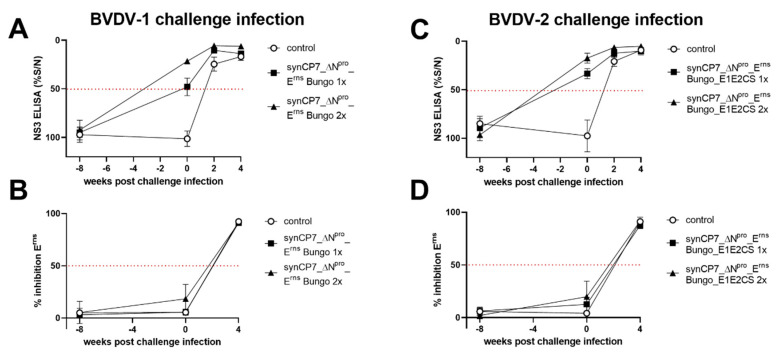
Serological determination of NS3- and E^rns^-specific antibodies after challenge infection. Serology was performed of BVDV-1b_synCP7_ΔN^pro^_E^rns^ Bungo-vaccinated and BVDV-1-challenged (**A**,**B**) or BVDV-1b_synCP7_ΔN^pro^_E^rns^ Bungo_E1E2CS-vaccinated and BVDV-2-challenged (**C**,**D**) cattle. The cattle were vaccinated with one (1x) or two (2x) shots of the candidate vaccine. (**A**,**C**) To monitor the serological response after vaccination and challenge infection, a competitive NS3 (p80) antibody ELISA was used. Relative blocking values (%S/N) are indicated as mean group values. (**B**,**D**) To determine the presence of E^rns^-specific antibodies, a competitive E0 (E^rns^) antibody ELISA was employed. Relative blocking values (% inhibition E^rns^) are indicated as mean group values. Error bars represent standard deviations. The dotted line marks the cut-off value of the test.

Vaccination was confirmed for all animals by the induced NS3 antibody titers, and the challenge infection boosted in addition the NS3 antibody titers of the vaccinated animal groups (Figure 7A,C). Furthermore, the presence of NS3-specific antibodies in the sera of all unvaccinated control animals at 14 days p.chall. was observed. Importantly, the commercially available competitive BVDV E0 (E^rns^) antibody ELISA could not detect any BVDV-E^rns^-specific antibodies after vaccination with either BVDV-1b_synCP7_ΔN^pro^_E^rns^ Bungo or BVDV-1b_synCP7_ΔN^pro^_E^rns^ Bungo_E1E2CS confirming the absence of BVDV-E^rns^ -specific antibodies and the applicability of the ELISA to discriminate BVDV-E^rns^ and BuPV-E^rns^-specific antibodies. In addition, the challenge infection induced BVDV-E^rns^-specific antibodies in both, the control and vaccinated groups (Figure 7B,D). All vaccinated animals seroconverted clearly for BVDV-E^rns^-specific antibodies within four weeks after challenge infection.

## 4. Discussion

Notably, different strategies have been pursued to develop effective marker vaccines for economically important animal pathogens. The marker principle is of great importance for the distinction of vaccinated and infected animals especially for disease control, eradication and pre-movement diagnostics for safe animal trade. As examples, a number of marker vaccines have been successfully developed and applied against BoHV-1, Suid herpesvirus 1 (SuHV-1, syn. Aujeszky’s disease virus (ADV) or pseudorabies virus (PrV)) and CSFV [23,59,60]. For BVD, the availability of a DIVA-compatible vaccine could contribute significantly to eradication programs that have been implemented in several countries worldwide [3]. However, a major issue in the development of a universal BVDV vaccine represents the high genetic and antigenic variability of the virus. The study of Yesilbag et al. has reported that the global distribution of BVDV subtypes varies within the continents, but BVDV-1b is a worldwide predominant subtype followed by BVDV-1a [35]. In Germany, the subtypes 1b and 1d were identified to be the dominant viruses in the samples collected from 2008–2016 [2]. BVDV-2 is more prevalent in Northern America and Asia, but a relevant percentage of cases has also been identified in Europe [12]. The outbreak of highly virulent BVDV-2c in 2012 in Germany was associated with severe clinical symptoms in infected cattle and resulted in mortality rates that varied between 2.3–29.5% in outbreak farms accompanied by major economic losses [61]. A combination of movement restrictions, disinfection, further biosecurity measures, and vaccination could prevent the further spread of BVDV-2c [2]. 

Overall, marker vaccine candidates should optimally induce immunity against both BVDV species. Thus, the aim was to develop a marker vaccine for BVDV-1 as well as for BVDV-2 using a new approach based on a synthetic backbone strategy with chimeric protein expression. For this purpose, chimeric BVD viruses were constructed on the genetic basis of the cytopathic BVDV-1b CP7 strain that contained E^rns^ of the atypical pestivirus BuPV. A BVDV-2 marker vaccine was generated by substitution of the genetic sequence of the BVDV-1 glycoproteins E1 and E2 by the heterologous BVDV-2 E1 and E2 of isolate CS8644. Furthermore, most of the IFN antagonist protein N^pro^ encoding sequence was deleted to generate a safe and highly attenuated modified live vaccine with limited replication capacities. Finally, BVDV-1b_synCP7_ΔN^pro^_E^rns^ Bungo and BVDV-1b_synCP7_ΔN^pro^_E^rns^ Bungo_E1E2CS were recovered from the respective infectious fully synthetic cDNA clones and characterized in vitro and in vivo. 

Safety and efficacy of the new potential marker vaccine candidates were tested in experimentally vaccinated calves that were subsequently challenged with a respective virulent heterologous BVDV-1 or -2 field strains. According to the prospective marker principle, vaccination of cattle with each of the vaccine candidates should induce seroconversion for BVDV E2- and NS3-specific antibodies while BVDV-specific E^rns^ -antibodies will only be detectable after BVDV challenge infection. 

The immunodominant proteins of pestiviruses are the glycoproteins E2 and E^rns^, and the nonstructural protein NS3. Neutralizing antibodies are predominantly produced against E2 [21] and only to a very minor extent against E^rns^ [21,62]. As far as known to date, the E^rns^ protein is unique to pestiviruses. It is part of the viral envelope but is also secreted in the supernatant of infected cell cultures [63], and cell-free E^rns^ is present in the circulation of persistently BVDV-infected cattle [27]. Furthermore, the E^rns^ protein displays RNase activity [64] with a preference for ssRNA [65] and is thereby involved in dampening of the IFN type I response of the host. The NS3 protein is a multifunctional protein that encompasses serine protease, helicase and NTPase activity [4]. In this study, the E^rns^ protein of BVDV-1 was substituted with the heterologous immunogenic E^rns^ protein of BuPV in the genetic backbone of the BVDV strain CP7 to produce a chimeric marker virus. Previous experiments had shown that the substitution of complete BVDV-E^rns^ with BuPV-E^rns^ resulted in a chimeric virus that showed a severe growth deficiency and could not be passaged more than two times in appropriate cells [37]. Therefore, the C-terminus of CP7 E^rns^, which harbors the transporter peptide region, was retained (aa 192–227) in the present study. In vitro growth kinetics revealed that the virus replication of BVDV-1b_synCP7_ΔN^pro^_E^rns^ Bungo was clearly impaired in comparison to BVDV-1b_synCP7_ΔN^pro^_E^rns^ Bungo_E1E2CS. The cleavage of E^rns^ and E1 is essential for the generation of infectious BVDV. It was shown that only one amino acid substitution in the C-terminus of E^rns^ may completely abolish the cleavage [66]. Therefore, it can be speculated that the sequences at the junction of BuPV-E^rns^ and CS-E1 (BVDV-2) might favor a more efficient processing of the viral polyprotein compared to the respective sequences at the transition of BuPV-E^rns^ and CP7-E1 (BVDV-1b). 

In addition to the exchange and modifications of the E^rns^ protein, most of the N^pro^ encoding sequences were deleted (nt 422–889). For CSF mutant viruses, the deletion of N^pro^ and abrogation of its immunosuppressive function led to an attenuation in vivo [67]. The attenuated BVDV vaccine Bovela^®^ (Boehringer Ingelheim Vetmedica GmbH, Ingelheim/Rhein, Germany) implemented both modifications introducing double individual genome mutations in the N^pro^ protease and E^rns^ RNase for attenuation into the strains KE9 (BVDV-1b) and NY93 (BVDV-2a) (citied from the CVMP assessment report for Bovela (EMEA/V/C/003703/0000)). Indicative for the IFN response in the host, IFN type I induction in infected cells was determined for the BVDV/BuPV chimeric viruses. All BVDV-1b_synCP7_ΔN^pro^ mutants were clearly impaired in their ability to prevent the induction of IFN type I (38–64-fold increase after 48 h p.i.) in comparison to the wild type viruses BVDV-1b_synCP7 (7.6-fold) and BuPV (1.2-fold; normalized to control). Enhanced in vitro replication of cp biotype viruses induces high levels of ssRNA and dsRNA compared to their corresponding ncp biotypes resulting in enhanced activation of IFN synthesis [33,68]. This could explain minor levels of IFN type I induction by the ncp BuPV in comparison to BVDV-1b_synCP7. In the context of BVDV-1b_synCP7_ΔN^pro^, further attenuation by substitution of the homologous E^rns^ by BuPV-E^rns^ was achieved. The chimeric virus BVDV-1b_synCP7_ΔN^pro^_E^rns^ Bungo showed a markedly impaired growth on KOR-R cells in combination with an enhanced induction of the IFN type I. The higher replication capacity of BVDV-1b_synCP7_ΔN^pro^_E^rns^ Bungo_E1E2CS may explain the better induction of type I IFN in comparison to BVDV-1b_synCP7_ΔN^pro^_E^rns^ Bungo. Combining the highly replicating CP7 strain with the deletion of N^pro^ and substitution of E^rns^ results in a disarmed virus that still efficiently induces IFN type I without any strategies left to circumvent the induction. This approach of generating IFN-sensitive viruses as attenuated virus vaccines has successfully been explored for a wide range of viruses, e.g., influenza virus [69], Ebola virus [70] and flaviviruses [71]. In contrast to the available modified live BVDV vaccine that exhibit the deletion of N^pro^ and the deletion of RNase activity of E^rns^, the described BVDV-1b_synCP7_ΔN^pro^_E^rns^ Bungo and BVDV-1b_synCP7_ΔN^pro^_E^rns^ Bungo_E1E2CS vaccine viruses are stronger attenuated indicated through lower level of replication [28]. The application of the cytopathic strain CP7 as the genetic background highly excludes the persistence of vaccine virus in the fetus. Both the generation of PI animals and the interference with ear notch-based control programs can be ruled out [42].

To estimate the genetic stability of the chimeric vaccines, serial passaging on IFN-competent cell cultures was conducted in vitro. Only three silent mutations for BVDV-1b_synCP7_ΔN^pro^_E^rns^ Bungo were found even after 20 passages in IFN-competent cells demonstrating a very high level of stability. No mutations were found for BVDV-1b_synCP7_ΔN^pro^_E^rns^ Bungo_E1E2CS after 10 passages. Furthermore, no virus could be re-isolated from nasal swabs or purified leukocytes from BVDV-1b_synCP7_ΔN^pro^_E^rns^ Bungo-vaccinated animals. In contrast, BVDV-1b_synCP7_ΔN^pro^_E^rns^ Bungo_E1E2CS vaccine virus could be re-isolated, but only from a single animal (#474) at only one time point from purified leukocytes (d 5) after the first vaccination, but the BVDV-2 vaccine was never re-isolated from nasal swabs. The safety of the vaccine viruses for pregnant dams has been additionally increased by using cp virus strains, as a reversion of cp to ncp BVDV strain in vivo is highly unlikely [72], which abrogates the possibility to induce persistent infection in the fetus as well as perpetuation within the cattle population. Overall, the application of cp virus strains in addition to the deletion of N^pro^ are cumulative safety measures that minimize the possibility of a persistent infection. MLVs based on cpBVDV have been shown to induce a strong humoral and cellular immunity and a solid fetal protection [5]. In contrast to subunit and nucleic acid vaccines that are considered to be safer, a single vaccination with MLVs can be sufficient to induce potent immunity [73]. Nevertheless, safety aspects need to be further investigated by vaccination of pregnant cows and in utero infection of growing fetuses to test for any possible induction of abortions. The very high level of attenuation makes any adverse effects highly unlikely and it can be assumed, that in intramuscularly vaccinated cows the vaccine virus candidates will not be able to reach the fetus.

In the present vaccination-challenge study, both chimeric vaccines reduced efficiently clinical symptoms in the bovine host. Body temperature elevation was clearly reduced, leukocytes counts decreased but recovered much faster, nasal virus shedding and cell bound viremia were significantly reduced in extent and in duration compared to the unvaccinated control groups. Twenty-one days after vaccination, all groups developed NS3-specific antibodies indicating effective replication of both chimeric viruses in the calves. Furthermore, the titers of neutralizing antibodies against the heterologous challenge strain ranged between 4 and 64 ND50 for both groups that were once vaccinated with either BVDV-1b_synCP7_ΔN^pro^_E^rns^ Bungo or BVDV-1b_synCP7_ΔN^pro^_E^rns^ Bungo_E1E2CS in comparison to the twice vaccinated groups with titers of 115 and 356 ND50, respectively. Previous studies have shown that neutralizing titers of 64 to 128 ND50 against the challenge strain prevented a systemic challenge virus infection [49]. The study showed that lower homologous titers of neutralizing antibodies were sufficient to confer a marked reduction of virus shedding and systemic replication in each of the vaccinated calves. Furthermore, the booster vaccination did not improve the antibody response or the clinical protection towards the challenge infection, irrespectively whether BVDV-1 or BVDV-2 was applied, suggesting that a single immunization is probably sufficient to induce protection against an infection in the described setting. The clinical parameters like rectal body temperature and the number of leukocytes were also indifferent in the once or twice vaccinated groups. The chimeric vaccines BVDV-1b_synCP7_ΔN^pro^_E^rns^ Bungo and BVDV-1b_synCP7_ΔN^pro^_E^rns^ Bungo_E1E2CS provided clinical protection against a high titer BVDV-1, and also a highly virulent BVDV-2 challenge infection, respectively. In a next step, it has to be evaluated whether the marked reduction of the challenge virus viremia is sufficient to induce a reliable and stable fetal protection. Furthermore, the combination of both chimeric viruses in a vaccine preparation and a consequently single immunization against both BVDV species should be investigated in the future. 

Finally, all vaccinated animals developed both NS3- and E2-specific antibodies, whereas no BVDV E^rns^-specific antibodies could be detected. These results showed that the vaccination success can be readily monitored and that a commercially available ELISA test can differentiate BuPV-E^rns^ and BVDV-E^rns^-specific antibodies without interference of cross-reactions. However, the challenge infection induced BVDV-E^rns^-specific antibodies in both, the control and vaccinated groups, confirming the applicability of the marker vaccine to discriminate vaccinated from BVDV-infected animals.

## 5. Conclusions

The availability of a DIVA-compatible BVD vaccine is an important pillar in disease eradication programs. The development of a chimeric marker vaccine is a new approach to serologically differentiate vaccinated from infected animals. Our study presents the first report on an efficient BVDV-1 and -2 modified live marker vaccine candidate and the accompanying commercially available serological marker ELISA system. The application of the presented chimera BVDV-1b_synCP7_ΔN^pro^_E^rns^ Bungo and BVDV-1b_synCP7_ΔN^pro^_E^rns^ Bungo_E1E2CS vaccines induced a robust immune response that protected from clinical disease after high titer challenge infections with virulent heterologous BVDV-1 and BVDV-2 field strains, respectively. Upon challenge infection, the vaccinated animals were protected from fever, experienced a shorter period of leukopenia followed by a strong rebound of leukocyte numbers. Most importantly, viremia as well as nasal shedding were significantly reduced in extent and in duration compared to the control groups. Interestingly, the number of vaccinations had no obvious influence on the protection status of the animals. The DIVA principle of the applied marker vaccine could be demonstrated using a commercially available BVDV-E^rns^-antibody assay, leading to a great improvement in the diagnostics of vaccinated animals and their differentiation from field-infected animals.

## 6. Patents

The patent “Pestivirus mutant for use in a vaccine” describes the deletion of N^pro^ in a cp BVDV and is active (EP2066342B1). The patent “Pestivirus marker vaccine” describes the mutant Pestivirus comprising a chimeric E^rns^ gene and is pending (EP3397280A1).

## Figures and Tables

**Table 1:** Virus isolation from nasal swabs and leukocytes after the BVDV-1 challenge infection. The cattle were vaccinated with one (1x) or two (2x) shots of the candidate vaccine. BVDV-susceptible KOP-R cell cultures were inoculated with four replicates of fluids from nasal swabs (A) and purified leukocytes (B) from EDTA-blood samples (100 µL per replicate). The virus replication was verified by immunofluorescence staining 3 days p.i.. Results were scored according to the number of positive inoculations out of the 4 replicates (0 = no BVDV isolation, 4 = all inoculations BVDV positive) and visualized using light (1) to dark grey (4) colored fields. A first result was confirmed after one blind passage of the supernatant of the first cell culture inoculation.

**(A) Virus Isolation from Nasal Swabs**																
**Group**	**Animal No.**	**Days post challenge infection**														**Cumulated**
		**1**	**2**	**3**	**4**	**5**	**6**	**7**	**8**	**9**	**10**	**11**	**12**	**13**	**14**	**Scoring values**
Unvaccinated	401	0	1	3	1	4	4	4	2	0	0	0	0	0	0	19
control	403	4	3	0	1	2	3	2	1	0	0	0	0	0	0	16
	407	1	4	2	1	1	1	2	2	0	0	0	0	0	0	14
	413	1	4	4	1	3	4	3	0	0	0	0	0	0	0	20
	876	0	4	1	3	1	4	2	1	0	0	0	0	0	0	16
BVDV-1b_synCP7_	406	0	0	0	1	0	0	0	0	0	0	0	0	0	0	1
ΔN^pro^_E^rns^ Bungo 1x	418	3	4	3	0	1	0	1	0	0	0	0	0	0	0	12
	893	1	0	0	0	0	0	0	0	0	0	0	0	0	0	1
	894	0	0	0	0	0	0	0	0	0	0	0	0	0	0	0
	898	0	0	0	0	3	0	0	0	0	0	0	0	0	0	3
BVDV-1b_synCP7_	405	0	0	0	0	1	0	0	0	0	0	0	0	0	0	1
ΔN^pro^_E^rns^ Bungo 2x	410	0	0	0	0	0	0	0	0	0	0	0	0	0	0	0
	412	0	0	0	0	1	0	0	0	0	0	0	0	0	0	1
	414	0	2	0	0	1	0	0	0	0	0	0	0	0	0	3
	417	0	0	0	0	3	0	0	0	0	0	0	0	0	0	3
**(B) Virus isolation from leukocytes**																
**Group**	**Animal No.**	**Days Post Challenge Infection**														**Cumulated**
		**1**	**2**	**3**	**4**	**5**	**6**	**7**	**8**	**9**	**10**	**11**	**12**	**13**	**14**	**Scoring Values**
Unvaccinated	401	0	0	1	1	4	4	3	2	1	1	0	0	0	0	17
control	403	0	2	0	1	2	4	2	1	0	0	0	0	0	0	12
	407	0	2	2	0	4	4	1	0	1	1	1	0	0	0	16
	413	0	4	1	4	4	4	3	1	0	0	0	0	0	0	21
	876	0	1	0	1	3	4	0	1	0	0	0	0	0	1	11
BVDV-1b_synCP7_	406	0	0	0	2	0	0	0	0	0	0	0	0	0	0	2
ΔN^pro^_E^rns^ Bungo 1x	418	0	0	0	0	3	0	0	0	0	0	0	0	0	0	3
	893	0	0	0	0	1	0	0	0	0	0	0	0	0	0	1
	894	0	0	1	0	0	0	0	0	0	0	0	0	0	0	1
	898	0	0	0	1	0	0	0	0	0	0	0	0	0	0	1
BVDV-1b_synCP7_	405	0	1	0	0	1	0	0	0	0	0	0	0	0	0	2
ΔN^pro^_E^rns^ Bungo 2x	410	0	0	0	0	3	1	0	0	0	0	0	0	0	0	4
	412	0	0	0	0	0	0	0	0	0	0	0	0	0	0	0
	414	0	0	0	1	1	0	0	0	0	0	0	0	0	0	2
	417	0	0	0	0	1	0	0	0	0	0	0	0	0	0	1

**Table 2:** Virus isolation from nasal swabs and leukocytes after the BVDV-2 challenge infection. The cattle were vaccinated with one (1x) or two (2x) shots of the candidate vaccine. BVDV-susceptible KOP-R cell cultures were inoculated with four replicates of fluids from nasal swabs (**A**) and purified leukocytes (**B**) from EDTA-blood samples (100 µL per replicate). The virus replication was verified by immunofluorescence staining at 3 days p.i.. Results were scored according to the number of positive inoculations out of the 4 replicates (0 = no BVDV isolation, 4 = all inoculations BVDV positive) and visualized using light (1) to dark grey (4) colored fields. A first result was confirmed after one blind passage of the supernatant of the first cell culture inoculation. nd = not determined.

**(A) Virus Isolation from Nasal Swabs**																
**Group**	**Animal no.**	**Days Post Challenge Infection**														**Cumulated**
		**1**	**2**	**3**	**4**	**5**	**6**	**7**	**8**	**9**	**10**	**11**	**12**	**13**	**14**	**Scoring Values**
Unvaccinated	478	0	0	0	2	4	4	4	3	3	0	4	0	0	0	24
control	480	0	0	2	0	3	3	4	4	1	0	0	0	0	0	17
	483	0	0	1	0	1	1	2	4	0	1	0	0	0	0	10
	490	0	0	0	0	2	4	1	nd	0	0	0	0	0	0	7
	492	0	0	1	0	0	4	4	2	0	0	0	0	0	0	11
BVDV-1b_synCP7_ΔN^pro^_	474	0	1	1	2	0	0	0	0	0	0	0	0	0	0	4
E^rns^ Bungo_E1E2 CS 1x	476	0	0	0	0	0	0	0	0	0	0	0	0	0	0	0
	477	0	0	0	0	0	0	0	0	0	0	0	0	0	0	0
	479	0	0	0	0	0	0	0	0	0	0	0	0	0	0	0
	487	0	0	0	0	0	0	0	0	0	0	0	0	0	0	0
BVDV-1b_synCP7_ΔN^pro^_	475	0	1	0	0	0	0	0	0	0	0	0	0	0	0	1
E^rns^ Bungo_E1E2 CS 2x	482	0	0	0	0	0	0	0	0	0	0	0	0	0	0	0
	484	0	0	0	0	0	0	0	0	0	0	0	0	0	0	0
	485	0	0	0	0	1	0	0	0	0	0	0	0	0	0	1
	489	0	0	0	0	0	0	0	0	0	0	0	0	0	0	0
**(B) Virus Isolation from Leukocytes**																
**Group**	**Animal no.**	**Days Post Challenge Infection**														**Cumulated**
		**1**	**2**	**3**	**4**	**5**	**6**	**7**	**8**	**9**	**10**	**11**	**12**	**13**	**14**	**Scoring Values**
Unvaccinated	478	0	3	2	4	4	4	4	4	2	3	1	0	0	0	31
control	480	0	3	2	4	4	4	4	4	1	0	1	0	0	0	27
	483	0	4	2	4	4	4	4	4	4	4	4	2	2	1	43
	490	0	3	0	1	4	3	3	1	0	1	0	0	0	0	16
	492	0	1	1	4	4	4	4	4	3	0	0	0	0	0	25
BVDV-1b_synCP7_ΔN^pro^_	474	0	0	2	1	1	0	0	0	0	0	0	0	0	0	4
E^rns^ Bungo_E1E2 CS 1x	476	0	0	1	1	3	2	0	0	0	0	0	0	0	0	7
	477	0	0	0	0	3	0	0	0	0	0	0	0	0	0	3
	479	0	0	0	1	0	0	0	0	0	0	0	0	0	0	1
	487	0	0	0	0	2	1	0	0	0	0	0	0	0	0	3
BVDV-1b_synCP7_ΔN^pro^_	475	0	0	0	1	2	0	0	0	0	0	0	0	0	0	3
E^rns^ Bungo_E1E2 CS 2x	482	0	0	1	2	2	1	0	0	0	0	0	0	0	0	6
	484	0	0	0	0	1	1	0	0	0	0	0	0	0	0	2
	485	0	0	0	0	3	1	0	0	0	0	0	0	0	0	4
	489	0	0	0	0	1	0	0	0	0	0	0	0	0	0	1
